# Hexarelin Signaling to PPARγ in Metabolic Diseases

**DOI:** 10.1155/2008/364784

**Published:** 2008-01-02

**Authors:** Annie Demers, Amélie Rodrigue-Way, André Tremblay

**Affiliations:** ^1^Research Center, Ste-Justine Hospital, University of Montreal, Montréal, PQ, Canada H3T 1C5; ^2^Department of Biochemistry, University of Montreal, Montréal, PQ, Canada H3T 1J4; ^3^Department of Obstetrics and Gynecology, University of Montreal, Montréal, PQ, Canada H3T 1C5

## Abstract

Investigating the metabolic functions of the nuclear receptor peroxisome proliferator-activated receptor γ (PPARγ) has been extremely rewarding over the past years. Uncovering the biologic roles of PPARγ and its mechanism of action has greatly advanced our understanding of the transcriptional control of lipid and glucose metabolism, and compounds such as thiazolidinediones which directly regulate PPARγ have proven to exhibit potent insulin-sensitizer effects in the treatment of diabetes. We review here recent advances on the emerging role of growth hormone releasing peptides in regulating PPARγ through interaction with scavenger receptor CD36 and ghrelin GHS-R1a receptor. With the impact that these peptides exert on the metabolic pathways involved in lipid metabolism and energy homeostasis, it is hoped that the development of novel approaches in the regulation of PPAR functions will bring additional therapeutic possibilities to face problems related to metabolic diseases.

## 1. INTRODUCTION

Vascular diseases impose the greatest burden upon health care systems and are predicted to remain the leading cause of death and disability in industrialized countries. The identification of excess body weight as a major risk factor, the epidemic of obesity and diabetes in Western societies and their increasing prevalence in children indicate that pathologies associated to the metabolic syndrome will continue to impact the health of individuals. Insulin resistance is a recurrent trait associated with increased adiposity, and despite the amplitude of health problems related to metabolic disorders, the mechanisms underlying excessive fat storage by adipocytes remain largely undefined.

The adipocyte is the major site of fatty acid storage in the body and plays a critical role in maintaining normal glucose and lipid homeostasis. If the capacity of the adipocyte to store lipids is exceeded, it can no longer regulate normally the release of fatty acids into the circulation, which ultimately leads to the abnormal accumulation of lipids in fat tissues and nonadipose depots. Such buildup of lipids in fat, liver, pancreatic islets, and muscle cells is associated to metabolic dysregulation of these tissues, resulting in many pathologic states of the metabolic syndrome, such as central obesity, atherosclerosis, type 2 diabetes, and insulin resistance [1, 2]. Over the recent years, with the unveiling of their ability to behave as master regulators of an array of genes that coordinate numerous pathways in lipid, glucose, and energy metabolism, the peroxisome proliferator-activated receptors (PPAR) have been considered important targets in the therapeutic management of metabolic disorders.

## 2. THE PPARs, FATTY ACID SENSORS

The PPARs consist of three isoforms, PPARα (NR1C1), PPARβ/δ (NR1C2), and PPARγ (NR1C3), all of which are *bona fide* members of the nuclear receptor family. Upon ligand activation, the PPARs act as transcription factors by directly binding DNA as obligate heterodimers with retinoid X receptor RXR (NR2B) to a peroxisome proliferator response element (PPRE) contained in the promoters of target genes. With identified ligands such as mono- and polyunsaturated fatty acids, and derivatives such as eicosanoids, the PPARs have been recognized as physiologic sensors for fatty acids that control the transcription of many genes governing lipid metabolism [3–5].

PPARα is predominantly expressed in the liver, where it activates a broad range of genes involved in fatty acid uptake, glycerol metabolism, β- and ω-oxidation of unsaturated fatty acids, and their transport into peroxisomes [6]. PPARα deficiency results in hypoglycemia and hypoketonemia, fatty liver, and elevated plasma fatty acids, revealing its importance in the hypoglycemic response [7, 8]. When fed a high-fat diet, PPARα-null mice are unable to catabolize fatty acids and develop severe hypertriglyceridemias without apparent obesity [9]. It is therefore predicted that fibrates, which selectively activate PPARα, are effective in treating hyperlipidemias [10]. PPARβ/δ is expressed ubiquitously and while biochemical and genetic evidence has linked PPARβ/δ to aspects of the metabolic syndrome [11–13], its emerging role in lipid metabolism remains to be further ascertained. Although the benefit of targeting PPARα and/or PPARβ/δ in lipid disorders is not excluded, the current review specifically emphasizes on PPARγ and its metabolic control by growth hormone releasing peptides.

## 3. PPARγ, A METABOLIC REGULATOR OF INSULIN RESISTANCE

Insulin resistance is marked by hyperinsulinemia, enhanced hepatic gluconeogenesis, and impaired insulin-stimulated glucose uptake into skeletal muscle and fat. Elevated levels of circulating fatty acids, associated with obesity and insulin resistance, increase fat accumulation in insulin target tissues and contribute to defective insulin action. In addition, obese adipose tissue-derived inflammation and altered secretion of adipocyte proteins, also known as adipokines or adipocytokines, can also impair insulin signals and affect systemic metabolism [14, 15]. The resulting hyperglycemia, dyslipidemia, and hypertension of the metabolic syndrome cause endothelial dysfunction and hasten vascular diseases.

Over the recent years, a number of adipokines, some of which being adipocyte-specific while others are not, have been identified to be produced and secreted by mature adipocytes. Adipokines, such as adiponectin and leptin which exhibit insulin-sensitizing effects, or resistin, tumor necrosis factor α(TNFα), and interleukin-6 (IL-6) which act as insulin resistance factors, all share autocrine, paracrine, or endocrine activity that regulates insulin sensitivity, therefore, establishing a role for the adipose tissue to function as an endocrine organ [14,16,17].

Remarkably, the thiazolidinediones (TZDs), which have been described as high-affinity ligands for PPARγ [18, 19], can modulate in a beneficial manner the expression of many if not all of these adipokines at the gene level, thereby correlating adipokine production with PPARγ activation. Originally discovered because of their potent insulin-sensitizing and glucose-lowering effects, TZDs are being used in clinics to correct abnormalities of lipid and glucose homeostasis, such as in type 2 diabetes, by reducing tissue insulin resistance [20]. For example, TZDs enhance adiponectin gene expression and circulating protein levels [21, 22], and decrease resistin [23, 24], TNFα [25], and IL-6 [26]. This suggests that the effect by which TZDs enhance insulin sensitivity likely resides in their ability to promote a beneficial profile of hormones secreted by adipocytes, which can then influence glucose disposal by the liver and muscle.

However, the mechanism by which TZD activation of adipocyte PPARγ leads to insulin sensitivity is not completely understood. Adipocyte-derived leptin is a circulating regulator of appetite and energy expenditure, whose increased levels reduce food intake and minimize ectopic lipid deposition by promoting fatty acid oxidation in peripheral tissues [27]. These effects contribute to the insulin-sensitizing properties of leptin, but its expression was found downregulated by PPARγ ligands [28, 29]. TZDs were also found to stimulate adipogenesis by upregulating many PPARγ target genes involved in fatty acid metabolism and storage [30]. Studies in rodent models and in humans have shown that TZD treatment causes weight gain [31, 32], an unwanted side effect that limits TZD efficacy on insulin sensitivity by increasing adiposity. This paradox remains largely unexplained, and among the likely hypotheses raised are a selective unequal accumulation of subcutaneous fat compared to visceral depots, and a possible activation of distinct yet overlapping adipogenic/antidiabetic gene programs in the adipocyte induced by TZDs [20, 33].

The use of genetic mouse models including tissue-specific deletion of the *Pparg* gene has enabled the identification of fat tissue as the primary target for TZDs but also revealed that other insulin-sensitive organs, such as liver and muscle, albeit expressing lower levels of PPARγ compared to fat, were also responsive to some extent to TZDs. Mice lacking white adipose fat, resulting in a phenotype similar to that of humans with lipoatrophic diabetes, fatty liver, hyperglycemia, and insulin resistance [31], or mice lacking adipose PPARγ, which also exhibit an insulin resistance phenotype [34], were refractory to the antidiabetic, but not the hypolipidemic effect of TZDs. In addition, these mice were highly predisposed to hepatic steatosis, an effect mainly attributed to liver PPARγ [35, 36]. TZDs also retained their glucose-lowering effects in liver- and muscle-specific PPARγ knockout mice [37, 38], arguying for a predominant role of adipose PPARγ in the insulin-sensitizing effects of TZDs, although another study reported that muscle PPARγ contributes to some extent to insulin resistance which was not improved by TZDs [39]. The kidney also appears as a target for TZDs in which however, renal PPARγ activation lead to fluid retention by inducing the Na^+^ transporter ENaC in the collecting duct [40, 41]. This adverse effect of TZDs is viewed as a serious complication for patients with preexisting congestive heart failure [42]. In addition, the prototype TZD troglitazone was withdrawn from clinics due to life-threatening hepatic toxicity, whereas the other two TZDs, rosiglitazone and pioglitazone, are still being used in large-scale clinical practice. Hence, the crucial benefit of TZDs to consistently lower fasting and postprandial glucose concentrations as well as free fatty acid concentrations in clinical studies is clearly established, but also tempered by other effects, mostly undesired, therefore adding complexity in our understanding of the systemic response to PPARγ ligands [43]. It thus becomes essential and of fundamental interest that other ways need to be identified in order to avoid the adverse effects of TZDs while keeping the benefits of correcting whole body glucose and fatty acid dysfunctions.

## 4. THE GHRP-PPARγ PATHWAY IN MACROPHAGES

One critical step initiating fatty streak formation in atherosclerosis consists in the accumulation of oxidized lipoprotein particles, mainly oxLDL, into the intima and their subsequent uptake by monocyte-derived macrophages, leading to the formation of cholesterol-loaded foam cells. Many lines of evidence suggest that the endocytosis of oxLDL by macrophages is mainly dependent upon their interaction with CD36, a member of the class B scavenger receptor family [44–47]. Studies in macrophages have shown that oxLDL uptake through CD36 provides a source of oxidized fatty acids and oxysterols that activate, respectively, PPAR and LXR (liver X receptor; NR1H3), thereby inducing a metabolic cascade resulting in enhanced expression of downstream genes, such as apolipoprotein E and ABC sterol transporters, and ultimately in cholesterol efflux to high density lipoproteins (HDL) [48]. However, these apparent beneficial effects are opposed by a positive feedback loop in which PPARγ activation by internalized fatty acids enhances the expression of CD36, a process shown to mediate foam cell formation [49–53].

CD36 is an 88 kDa glycoprotein originally identified as a platelet receptor and also known as fatty acid translocase, which is expressed in numerous cell types including monocytes/macrophages, platelets, endothelial cells, and adipocytes [53–55]. CD36 is a multiligand receptor that is recognized by fatty acids, anionic phospholipids, thrombospondin, and oxidized lipoproteins. It is this latter property of scavenging (e.g., clearing) oxLDL which implicates CD36 in the initial steps of atherogenesis, as evidenced with studies in mice [53, 56] and humans [57].

The findings that growth hormone releasing peptides (GHRPs) serve as ligands for CD36 [58, 59] led to the evaluation of their potential role in regulating cholesterol metabolism in macrophages. The GHRPs belong to a class of small synthetic peptides known to stimulate growth hormone release through binding to the GH secretagogue-receptor 1a (GHS-R1a), a G-protein-coupled receptor originally identified in hypothalamus and pituitary [60] and later recognized as the receptor for ghrelin [61]. The peripheral distribution of the ghrelin GHS-R1a receptor in tissues, such as heart, adrenals, fat, prostate, and bone, has supported physiological roles of ghrelin and GHRPs not exclusively linked to GH release. For example, GH-independent effects on orexigenic properties, fat metabolism, bone cell differentiation, and hemodynamic control have been reported for ghrelin and GHRPs [62, 63]. Also, in conditions in which GH release was not promoted or in GH-deficient animals, the GHRP hexarelin was shown to feature cardioprotective effects by preventing ventricular dysfunction [64, 65], and by protecting the heart from damages induced by postischemic reperfusion [66]. These studies suggest that part of the beneficial effects of hexarelin may not involve GH release.

To evaluate the potential of hexarelin to regulate cholesterol metabolism in vivo, apolipoprotein E (apoE)-null mice maintained on a long-term high-fat and high-cholesterol diet, a condition known to promote atherosclerosis, showed a significant regression in plaque formation when treated with hexarelin compared to saline-treated controls [67]. These beneficial effects were observed in conditions in which GH was not upregulated by hexarelin [67], and also using EP80317, an hexarelin derivative with no GH release activity [68], supporting a GH-independent role for GHRPs.

**Figure 1:**
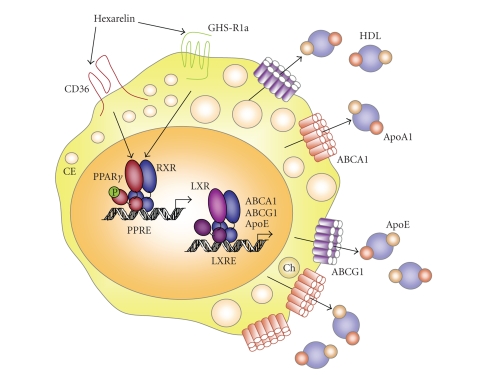
*A GHRP-PPAR* γ *pathway in macrophages.* Overview of the effects of hexarelin which by interacting with scavenger receptor CD36 and GHS-R1a ghrelin receptor promotes the transcriptional activation of PPARγ. LXRα which is a target of PPARγ is then upregulated with the subsequent increase in apolipoprotein E (apoE) and sterol transporters ABCA1 and ABCG1 expression. Activation of the PPARγ-LXRα-ABC metabolic pathway in response to hexarelin favors cholesterol efflux by macrophages through high density lipoproteins (HDL). Adapted from [52].

To address the mechanism by which hexarelin exerts these beneficial effects, treatment of differentiated THP-1 macrophages or mouse peritoneal macrophages with hexarelin resulted in an increase in cholesterol efflux, which correlates with an enhanced expression of LXRα, apoE, and sterol transporters ABCA1 and ABCG1, all involved in promoting the high density lipoprotein (HDL) pathway (see Figure 1). In addition, these effects were severely impaired in treated peritoneal macrophages isolated from PPARγ heterozygote mice, implying an essential role for PPARγ in mediating the response to hexarelin [67]. We further showed using cell reporter assays that the interaction of hexarelin with CD36 or with ghrelin receptor resulted in an enhanced transcriptional activation of PPARγ, suggesting that both receptors signal to PPARγ [67]. These studies have helped to define that the beneficial effects of hexarelin involved the activation of the PPARγ-LXRα-ABC metabolic cascade, thereby causing macrophages to mobilize excess cholesterol into the HDL cholesterol reverse pathway [67]. These findings therefore support a novel regulatory pathway by which CD36 and possibly ghrelin receptor may impact PPARγ-regulated functions. Consequently, a detailed knowledge of the concerted modulation of CD36 and ghrelin receptor signaling pathways may help to provide additional strategies in pathologic conditions such as atherosclerosis.

## 5. A GHRP-PPARγ PATHWAY IN ADIPOCYTES

Based on our observations that hexarelin promotes PPARγ activation through CD36 and ghrelin receptors in macrophages [67], we wanted to address whether hexarelin could exert activation of PPARγ and subsequent downstream effects in adipocytes. PPARγ is considered a master regulator of fatty acid metabolism in fat through its direct role in regulating the expression of a broad range of genes involved in fatty acid and glucose metabolism. Among the genes upregulated by PPARγ are found genes related to fatty acid uptake (fatty acid transport protein FATP, CD36), glucose uptake (GLUT4), β-oxidation (acyl-CoA dehydrogenase, carnitine palmitoyltransferase CPT-1, acyl CoA oxidase), gluconeogenesis (phosphoenolpyruvate carboxykinase PEPCK), and lipid storage (adipophilin) ([69, 70], and references therein). Increased expression of many of these genes might result in a net influx and trapping of fatty acids into adipocytes, which is considered a mechanism by which TZDs consistently reduce circulating free fatty acids.

Mature adipocytes are known to express CD36 but not the other hexarelin receptor GHS-R1a ([71, 72], and data not shown). Whereas the role of CD36 in mediating oxLDL-derived cholesterol and fatty acid uptake by macrophages is recognized, the mechanisms by which CD36 may impact the overall metabolic activity of fat storage and mobilization by adipocytes is not completely understood. With these considerations and the central role of PPARγ in regulating many aspects of fatty acid metabolism, it was expected that hexarelin may impact PPARγ-regulated events in adipocytes.

**Figure 2:**
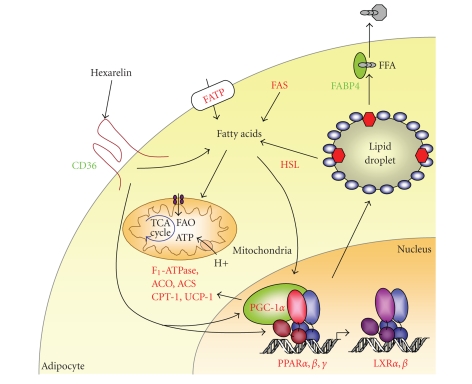
*Hexarelin promotes mitochondrial activity in adipocytes.* Scheme of gene expression analysis of fatty acid metabolic regulators in 3T3-L1 adipocytes. Shown are a subset of genes identified as upregulated (red) or downregulated (green) by hexarelin compared to untreated cells. These effects of hexarelin require CD36 which is expressed in adipocytes as opposed to GHS-R1a receptor; FAO, fatty acid oxidation; FABP, fatty acid binding protein; FAS, fatty acid synthase; HSL, hormone-sensitive lipase; ACO, acyl CoA oxidase; ACS, and acyl CoA synthase. Other abbreviations appear in text.

As such, we recently reported the ability of hexarelin to regulate PPARγ-dependent downstream events in cultured adipocytes and in fat tissues from treated mice [73], thereby providing evidence that hexarelin may target different PPARγ expressing tissues. In these studies, we observed that treatment of differentiated 3T3-L1 adipocytes with hexarelin resulted in a depletion in triglyceride cellular content, accompanied by profound changes in the gene expression profile of key markers of fatty acid metabolism [73]. Interestingly, many of these genes were shared with TZD troglitazone treatment, indicating that PPARγ may be considered as a common regulator in both responses. Consistent with this, among the genes upregulated by hexarelin, we found many established PPARγ targets, such as nuclear receptor LXRα, FATP1 (fatty acid transport protein), and F_1_-ATP synthase (see Figure 2). Other genes involved in various aspects of entry, transport, synthesis, and mobilization of fatty acids, such as hormone-sensitive lipase (HSL), fatty acid synthase (FAS), and acetyl-CoA synthase (ACS) among others, were also upregulated, whereas glycerol-3-phosphate acyltransferase (GPAT), which catalyzes the initial and committing step in glycerolipid biosynthesis, was downregulated by hexarelin [73]. All together, this type of profile is strongly suggestive of an increase in the cellular mobilization of free fatty acids in response to hexarelin.

However, the response to hexarelin was not totally mimicked by troglitazone as other described PPARγ targets, such as adipocyte fatty acid binding protein FABP4 (also referred to as aP2) and lipid droplet-associated protein adipophilin remained mostly unchanged upon treatment with hexarelin [73]. It is also important to note that gene expression and protein levels of CD36, a well-known target of PPARγ [49, 50], were not changed by hexarelin, as opposed to troglitazone which significantly induced both in treated adipocytes. Similar results were also found in macrophages, indicating that this regulation is not cell-specific [67], and may prevent any undesired increase in macrophage CD36, a situation that correlates with proatherosclerotic events [55, 74]. Also, as opposed to troglitazone which decreased PPARγ expression, hexarelin contributed to maintain expression and steady-state levels of PPARγ in adipocytes and macrophages [67, 73]. The exact mechanism(s) by which hexarelin exerts such gene-specific regulation compared to TZDs are not clearly understood, but differences in PPARγ occupancy of targeted promoters and/or posttranslational modifications of PPARγ are certainly among the likely possibilities to consider in the response of PPARγ to hexarelin ([67], see below).

## 6. HEXARELIN PROMOTES MITOCHONDRIAL ACTIVITY AND BIOGENESIS

Uptake of fatty acids and glucose by muscle and fat tissues is an important component regulating energy expenditure and defects in CD36 have been associated with impaired fatty acid and glucose homeostasis in humans [75, 76]. However, the role of CD36 in regulating energy metabolism in adipocytes remains an unresolved issue.

By transposing the ability of hexarelin to promote PPARγ activation to adipocytes, it was interesting to observe that many genes upregulated by hexarelin were characteristic of an enhanced profile of fatty acid oxidation and mitochondria morphology [73]. More specifically, among the genes upregulated were found acetyl CoA acyl transferase, CPT-1, and several subunits of the ATP synthase and of the cytochrome c oxidase complexes, all suggesting an increased fatty acid mobilization towards the mitochondrial oxidative phosphorylation pathway [73].

Enhanced mitochondrial oxidative potential is required to supply adequate ATP production in high energy-demanding processes, such as adaptation to cold in brown fat, heart and skeletal muscle contraction, and liver gluconeogenesis in response to fasting. Such mitochondrial energy-producing capacity correlates with active β-oxidation of fatty acids and increased expression of PPARγ coactivator-1 (PGC-1) in these tissues [77–82]. PGC-1α is a coactivator of most nuclear receptors that was discovered as a molecular switch that turns on several key components of the adaptive thermogenic program in brown fat, including the stimulation of fuel intake, mitochondrial fatty-acid oxidation, and heat production [83, 84]. These metabolic changes are supported by the ability of PGC-1 to upregulate the expression of UCP-1, a biological uncoupler of mitochondrial oxidative phosphorylation, and of genes of gluconeogenesis, such as PEPCK and glucose-6-phosphatase (reviewed in [84, 85]). Thus, modulating the relative activity of PGC-1 within a particular tissue may lead to a fine tuning of mitochondrial function in fatty acid oxidation and energy balance. Interestingly, hexarelin induced an increase in PGC-1α and UCP-1 in 3T3-L1 adipocytes as well as in epididymal fat of treated mice, indicating a potential fat burning phenotype taking place in white fat in response to hexarelin [73]. Consistent with these changes, electron microscopy of hexarelin-treated 3T3-L1 adipocytes showed an intense and highly organized cristae formation that spans the entire width of mitochondria compared to untreated cells, accompanied with an increase in cytochrome c oxidase activity, two features characteristic of highly oxidative tissues [73]. A similar mitochondrial phenotype and gene expression profile was detected in epididymal white fat of mice treated with hexarelin, and shown to be dependent on CD36, indicating that the ability of hexarelin to promote a fat burning-like phenotype was maintained in vivo [73]. These studies therefore support a functional GHRP-PPARγ signaling cascade in adipocytes, which provides a potential role for CD36 to impact the overall metabolic activity of fatty acid usage and mitochondrial biogenesis in fat. These aspects are particularly relevant to the emerging association of mitochondrial dysfunction with insulin resistance and type 2 diabetes [86].

## 7. HEXARELIN INCREASES PPARγ PHOSPHORYLATION

The exact mechanism(s) by which PPARγ activity is modulated in response to hexarelin remains to be clearly defined. In an attempt to partly characterize such a response, we found that PPARγ was highly phosphorylated in macrophages treated with hexarelin, therefore providing a basis on how PPARγ can respond to hexarelin signaling [67]. Although macrophages do express both receptors recognized by hexarelin, our observation that GHS-R1a activation by hexarelin enhanced PPARγ activity in transfected heterologous cells may therefore suggest that GHS-R1a signals to activate PPARγ [67]. Consistent with this, the activation of GHS-R1a receptor by hexarelin or its natural ligand ghrelin leads to the phosphorylation of PPARγ in macrophages, while a GHRP selective for CD36 did not ([67] and unpublished observations). These findings rather implicate GHS-R1a signaling in the phosphorylation of PPARγ, at least in macrophages.

The effects of phosphorylation on PPARγ activity have been reported to vary, often in opposite directions, depending on the cellular and promoter context [87]. In that respect, it is interesting to note that while PPARγ ligands of the TZD family are recognized to upregulate CD36 gene expression [49, 50], no significant changes in CD36 expression were measured in response to GHRPs despite PPARγ activation [67,68,73]. In order to further investigate the mechanism by which this unexpected regulation of CD36 by hexarelin may result, chromatin immunoprecipitation assay has revealed that the relative occupancy of the CD36 promoter region by PPARγ remained mostly unchanged, whereas that of nuclear receptor LXRα, also a known target of PPARγ [88], was occupied by PPARγ in a greater extent in macrophages treated with hexarelin, indicating that LXRα upregulation by hexarelin may result from a preferred recruitment of PPARγ to the LXRα promoter, as opposed to CD36 [67]. Whether PPARγ phosphorylation may discriminate for promoter usage is not yet known but interestingly, it was reported that PPARγ phosphorylation could decrease CD36 transcription in macrophages [53]. Given the ability by which posttranslational modifications such as phosphorylation could regulate PPARγ transcriptional activity and that ligand-independent recruitment of transcriptional coregulators is favored by nuclear receptor phosphorylation [87 89–91], it is predicted that such mechanism may contribute in the cellular response to hexarelin by selectively regulating PPARγ-targeted genes. These aspects need to be further investigated in order to ascertain such selectivity.

## 8. CONCLUDING REMARKS

Although the exact mechanisms by which GHRPs promote their metabolic response are not fully understood, it becomes clear that interacting with CD36 and/or GHS-R1a receptors induces profound changes in metabolic activities of target tissues, especially regarding PPARγ-regulated events. However, it is important to note that the sole activation of PPARγ may not be exclusive in translating the signal by hexarelin or other GHRPs. Indeed, in view that hexarelin can also promote PPARα and PPARβ/δ activation [67], and with the propensity of PGC-1α to coactivate other nuclear receptors besides PPARγ, such as thyroid hormone receptor TRα, retinoic acid receptor RARα, estrogen-related receptor ERRs, and PPARα [83], it is expected that these pathways may also be affected by hexarelin. So clearly, the mechanism(s) by which hexarelin exerts its metabolic effects represents a promising avenue which deserves further investigation to face problems related to multipathological states associated with metabolic syndrome.
